# Successful Multidisciplinary Repair of Severe Bilateral Uretero-Enteric Stricture with Inflammatory Reaction Extending to the Left Iliac Artery, after Robotic Radical Cystectomy and Intracorporeal Ileal Neobladder

**DOI:** 10.3390/curroncol29010014

**Published:** 2021-12-29

**Authors:** Mariangela Mancini, Alex Anh Ly Nguyen, Alessandra Taverna, Paolo Beltrami, Filiberto Zattoni, Fabrizio Dal Moro

**Affiliations:** 1 Urological Clinic, University Hospital of Padova, 35121 Padova, Italy; alexanhly.nguyen@gmail.com (A.A.L.N.); alessandra.taverna3@gmail.com (A.T.); paolo.beltrami@aopd.veneto.it (P.B.); filiberto.zattoni@unipd.it (F.Z.); fabrizio.dalmoro@unipd.it (F.D.M.); 2 Department of Surgical, Oncological and Gastroenterological Sciences, University of Padova, 35121 Padova, Italy

**Keywords:** uretero-enteric strictures, robotic radical cystectomy, urinary diversion, surgical oncology, vascular reconstruction, ureteroneocystostomy, bladder cancer

## Abstract

Uretero-enteric anastomotic strictures (UES) after robot-assisted radical cystectomy (RARC) represent the main cause of post-operative renal dysfunction. The gold standard for treatment of UES is open uretero-ileal reimplantation (UIR), which is often a challenging and complex procedure associated with significant morbidity. We report a challenging case of long severe bilateral UES (5 cm on the left side, 3 cm on the right side) after RARC in a 55 years old male patient who was previously treated in another institution and who came to our attention with kidney dysfunction and bilateral ureteral stents from the previous two years. Difficult multiple ureteral stent placement and substitutions had been previously performed in another hospital, with resulting urinary leakage. An open surgical procedure via an anterior transperitoneal approach was performed at our hospital, which took 10 h to complete, given the massive intestinal and periureteral adhesions, which required very meticulous dissection. A vascular surgeon was called to repair an accidental rupture that had occurred during the dissection of the external left iliac artery, involved in the extensive periureteral inflammatory process. Excision of a segment of the external iliac artery was accomplished, and an interposition graft using a reversed saphenous vein was performed. Bilateral ureteroneocystostomy followed, which required, on the left side, the interposition of a Casati-Boari flap harvested from the neobladder, and on the right side a neobladder-psoas-hitching procedure with intramucosal direct ureteral reimplantation. The patient recovered well and is currently in good health, as determined at his recent 24-month follow-up visit. No signs of relapse of the strictures or other complications were detected. Bilateral ureteral reimplantation after robotic radical cystectomy is a complex procedure that should be restricted to high-volume centers, where multidisciplinary teams are available, including urologists, endourologists, and general and vascular surgeons.

## 1. Introduction

Radical cystectomy (RC) with pelvic lymph node dissection represents the standard of care in muscle-invasive or refractory non-muscle-invasive bladder cancer. Recently, robot-assisted RC (RARC) has become more common. However, the operation remains a challenging procedure with significant complications.

Uretero-enteric anastomotic strictures (UES) represent the main cause of renal dysfunction after urinary diversion (UD) [1] and can result from compromised vascularity (i.e., previous irradiation or excessive ureteral dissection) or improper surgical technique [2,3]. The gold standard for treatment of UES is open uretero-ileal reimplantation (UIR) [4,5,6]. However, open revisions in such cases are complex procedures, associated with significant morbidity [4]. Robotic repair of UES has been described, with good results, in limited selected cases [7,8,9].

## 2. Case Report

### 2.1. Patient History

In June 2017, a 55 years old heavy smoker male patient was diagnosed with transitional cell carcinoma of the bladder (T2HG + Cis, after TURB) and submitted, at another institution, to RARC with intracorporeal ileal neobladder, following neo-adjuvant chemotherapy (Gemcitabine + Cisplatin).

**Figure 1:**
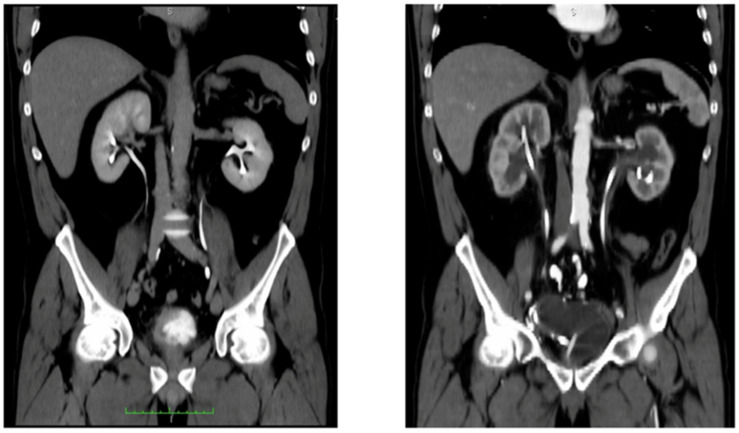
(**Left**) CT-urography showing pathological bladder thickening, later diagnosed as MIBC, with regular upper urinary tracts. (**Right**) CT scan, arterial phase, taken after development of bilateral ureteral-ileal stenosis with hydronephrosis treated at the previous institution with ureteral stentin. Green scale bar: cms.

The surgical technique at the previous institution included the selection of a 42 cm ileal segment for the neobladder (Vescica Ileale Padovana or Padua Ileal Neobladder [10]), the passage of ureters through the posterior neobladder wall, and bilateral ureteroileal anastomoses according to the modified split-nipple technique, with double-J (DJ) stents [11]. The pathology revealed a transitional cell carcinoma of the bladder, pT1 + Cis, pN0 (47/47 lymph nodes were negative). The DJ stents were removed after three months. After stent removal, acute kidney insufficiency and bilateral hydroureteronephrosis developed, until the uretero-enteric anastomoses required the placement of bilateral percutaneous nephrostomies; after a month, these were removed and converted to ureteral bilateral DJ stents, which were changed after six months (Figure 1). In December 2018, an attempt to remove the stents was made, but bilateral hydroureteronephrosis developed again, more severely on the left side. The bilateral nephrostomies were re-positioned. A transnephrostomic radiologic evaluation demonstrated bilateral stenosis of the uretero-enteric anastomosis, which was more severe on the left side. DJ stents were bilaterally placed in an anterograde fashion. The right nephrostomy was removed after a week; the left one was removed after two months. The stents were kept in place for the following 18 months at the previous institution, with stent-changing becoming gradually more challenging and with signs of initial renal function deterioration (the serum creatinine after two years was 204 umol/L). At this point, the patient was referred to our attention. He presented good day- and night-time continence and no significant urinary tract symptoms or post-urination residue.

### 2.2. Treatment

**Figure 2:**
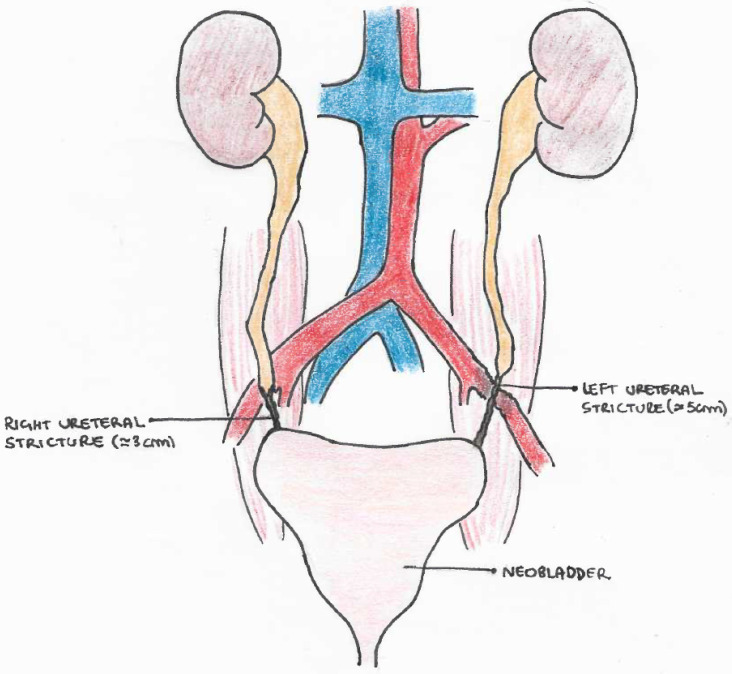
Drawing of bilateral UES in intracorporeal neobladder (VIP), with involvement of left external iliac artery in the scarring process.

At the initial evaluation, the neobladder showed a good capacity of 400 cc. A retrograde ureteropielography documented a bilateral stenosis of the terminal ureters, extending upwards for 3 cm on the right side and 5 cm on the left side, where the left distal ureter appeared as a filiform string (Figure 2).

**Figure 3:**
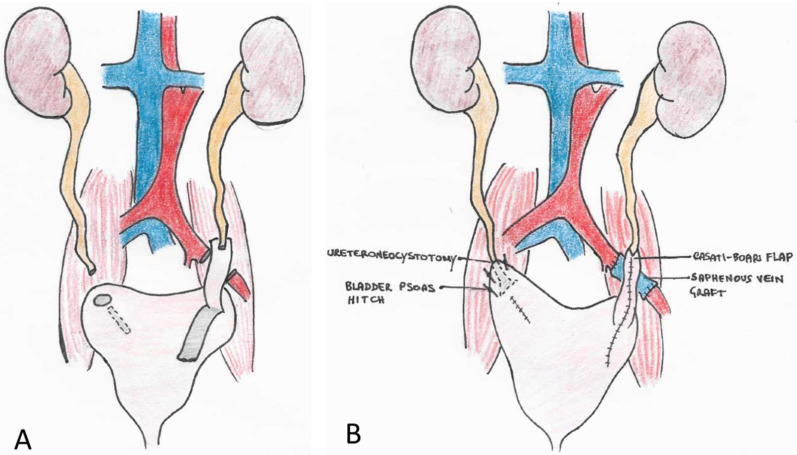
Arterio-arterial saphenous vein external iliac bypass plus resection of the bilateral UES, on the left side with the interposition of a Casati-Boari flap. (**A**) demolition phase; (**B**) reconstructive phase.

After admission to our hospital, surgery was planned and the DJ stents were removed, with bilateral positioning of percutaneous nephrostomies in an attempt to reduce ureteral wall inflammation before surgery. The patient was temporarily discharged. The preoperative CT scan showed no signs of cancer, except for a small 1.5 cm tumor at the upper lobe of the right lung, which had been stable since 2018 and for which treatment was deferred. One month later, an abdominal open surgery procedure through an anterior transperitoneal approach was performed. The operation required 10 h to complete. A supraumbilical-pubic incision was made, with careful dissection of massive intestinal adhesions. The retroperitoneum, especially on the left side, was highly fibrotic. The left ureter was found to be tenaciously adherent to the upper sigmoid and the left external iliac artery, surrounded at this level by a thick inflammatory tissue that made the dissection extremely challenging. A vascular surgeon was called to repair the external left iliac artery during the dissection, which had resulted in a small effraction of the artery that was easily controlled without significant blood loss. Since the arterial wall was diffusely malacic and involved in the inflammatory process, direct reconstruction of the vessel was not attempted. Excision of a segment of the left external iliac artery was accomplished, after proximal and distal arterial clamping, and an interposition graft was inserted using a reversed right saphenous vein segment taken from the patient’s right upper thigh. Resection of the ureteral strictures and bilateral ureteroneocystostomy followed, which required, on the left side, the interposition of a 6 cm Casati-Boari flap, harvested from the neobladder (VIP). The Casati-Boari flap was placed directly in front of the saphenous vein graft. On the right side, a bladder-psoas hitching technique was possible, with direct intramucosal reimplantation of the ureter in the posterior face of the neobladder, in the absence of tension (Figure 3). Intraoperative total blood loss was 1000 mL.

**Figure 4:**
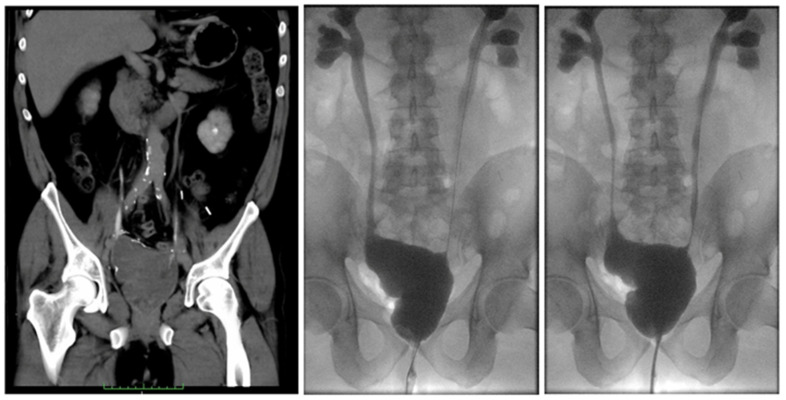
Outcome after open surgical correction of the stenosis in our hospital. (**Left**) CT-urography. (**Center** and **right**) cystography and bilateral retrograde ureteropylography images at different time points. Green scale bar: cms.

The patient’s perioperative outcomes are summarized in Table 1. A bladder catheter and 6 Ch MJ stents were left in place at the end of the surgery. Serum creatinine (preoperative value: 204 umol/L) was 178 umol/L on the first postoperative day, and dropped to 136 umol/L on the seventh day and 125 umol/L on the fourteenth day. The nephrostomy tubes were initially left open in place, and were closed on the tenth postoperative day. Seven days later, after opening of the nephrostomies, with no evidence of residual urine in the kidneys, we first removed the right nephrostomy and then, after 24 h, the left one (as it is the custom in our clinic to remove nephrostomies while the stents are still in place to favor the closure of the nephrostomy flank openings). However, we observed leakage of urine from the abdominal drain on the left side after removal of the left nephrostomy. We performed a retrograde pyelogram, which showed leakage from the left uretero-ileal anastomosis (at the Casati-Boari flap and the left ureter anastomosis site). We positioned bilateral 6 Ch MJ stents, without resolution of the urinary leakage from the abdominal drain. Three days later, the stents were substituted with bilateral larger ones, 8 Ch, with urinary leakage resolution. Serum creatinine on the twenty-first postoperative day was 137 umol/L. The MJ stents were left in place for ten additional days, and then changed to DJ stents. The patient was discharged on the 28h postoperative day, with a serum creatinine of 140 umol/L. The urinary catheter was removed at the patient’s home after ten days. After catheter removal, the patient resumed spontaneous per uretram micturition, with good preserved urinary continence. The pathology report of the excised distal ureteral tracts showed intestinal glandular metaplasia of the distal ureters and signs of severe inflammatory reaction at the level of the periureteral tissue, extending on the left side to the left external iliac artery wall, with signs of severe ulceration. Two months later, the patient was readmitted to our hospital and the lung tumor was removed via a thoracoroscopic lobectomy (pathology: adenocarcinoma of the lung, pT2aN0). Two days later, the DJs were removed after radiological confirmation (cystography and bilateral ureteropielography) of a good surgical outcome (Figure 4). The patient resumed natural micturition per uretram with good continence and without significant residue. At the 6-, 9-, 12-, and 24-month follow-up visits, the patient was in good health, with serum creatinine stable at 140 umol/L. No signs of cancer or of relapse of the strictures, or other complications, were detected.

## 3. Discussion

UES following UD occur in 3–10% of patients after RC [5]. They can cause irreversible disability, such as chronic kidney insufficiency. Debate regarding the incidence of UES after open or robotic RC is ongoing, without definitively proven differences between surgical approaches [12,13]. Moreover, in cases of RARC, the different incidence of UES after ICUD or ECUD remains unclear, given the lack of randomized prospective studies [3]. Patients with previous urinary leakage at the uretero-enteric anastomotic site or previous episodes of UTIs, with impaired healing and scarring caused by the inflammatory mediators and released proteases, show a much higher incidence of UES [8]. UES repair after RC represents a challenging surgical procedure, performed in morbid patients who have often submitted previously to multiple endourological procedures with urinary leakage, resulting in severe inflammation of the ureters and the retroperitoneum. In this scenario, the risk of complications such as blood loss, bowel perforation, or vascular involvement is high [3,4]. Currently, there are no established standardized therapies for treatment of UES after RC. Different cases must be managed in different ways, and no rigid options should be adopted [3]. Although endourologic treatment represents a minimally invasive first option to consider, it shows a low success rate compared to surgical repair, especially when the stricture length is longer than 1 cm [4]. Once surgery is planned, either open repair or robotic repair is possible. Open UIR is the gold standard treatment, offering excellent success rates, especially in particularly complex cases involving different organs [3]. Recently, robotic repair has been proposed as a safe option in the management of UES after RARC [7,13]. A single-port platform has been successfully introduced in three cases [14]. However, evidence is scarce on this topic, particularly in cases of bilateral severe strictures longer than 3 cm. Further studies are required, focused particularly on cases of high complexity [3].

## 4. Conclusions

Surgical correction of UES after RARC and intracorporeal neobladder is feasible and effective but can present numerous hurdles to overcome and requires high surgical skills and often multidisciplinary involvement. Robotic correction has been described in some reports, but an open surgical approach can be preferred in more severe cases where the abdominal scarring process is advanced and the length of the stenosis is longer than 3 cm. An open mindset should be maintained, selecting different options in different cases. The more complex procedures should be restricted to high-volume centers, where multidisciplinary teams are available to support each other, including urologists, endourologists, and general and vascular surgeons.

## Figures and Tables

**Table 1:** Perioperative patient outcomes.

	Day 0	Postoperative Day 1	Day 7	Day 14	Day 21	Day 28
***Stricture length (cm)***	Left: 5, Right 3					
***Operative time (hours)***	10					
***Estimated blood loss (ml)***	1000					
***Serum creatinine (umol/L)***	204	178	136	125	137	140
***Diuresis (ml)***	3500	2200	3000	2500	2000	2500
***Temperature (°C)***	37.5	37.3	36.5	36.3	36.5	36.6
***Hb (g/dL)***	9.0	9.1	9.4	9.5	9.9	11
***Complication***	-	-	-	Urinary leak,left	-	-
***Hospital stay***						Hospital discharge

## Data Availability

Data are contained in the article. Additional data are available on request from the corresponding author.
